# The association between sexual orientation, BMI, obesity diagnosis, and provider recommendation for weight management

**DOI:** 10.1186/s12905-021-01585-x

**Published:** 2022-01-26

**Authors:** Kristen M. Wolfgang, Junko Takeshita, Robert Fitzsimmons, Carmen E. Guerra

**Affiliations:** 1 University of Pennsylvania Perelman School of Medicine, 3400 Civic Center Blvd, Philadelphia, PA 19104 USA; 2 Department of Dermatology and Department of Biostatistics, Epidemiology and Informatics, University of Pennsylvania Perelman School of Medicine, 3400 Civic Center Blvd, Philadelphia, PA 19104 USA; 3 Department of Dermatology, University of Pennsylvania Perelman School of Medicine, 3400 Civic Center Blvd, Philadelphia, PA 19104 USA; 4 Division of General Internal Medicine, University of Pennsylvania Perelman School of Medicine, 423 Guardian Drive, Philadelphia, PA 19104 USA

**Keywords:** Obesity, Overweight, Lesbian, Bisexual, Healthcare disparities, LGBTQ, Preventive health

## Abstract

**Background:**

National data show that lesbian and bisexual women are more likely to be obese compared to straight women. However little is known about whether provider recommendation for weight management varies across these populations. Furthermore, health care providers have explicit and implicit preferences for straight people in comparison to lesbian or gay people. There is little research that exists depicting how this preference affects quality of patient care. The purpose of the study is: to compare, among lesbian, bisexual, and straight females with BMIs ≥ 30: (1) the average Body Mass Index (BMI); (2) receipt of a diagnostic code for obesity; and (3) receipt of a provider recommendation for weight management.

**Methods:**

We performed a cross-sectional study of 534 patient records from four outpatient academic internal medicine practices at the University of Pennsylvania between January 1, 2019 to December 31, 2019 to determine variations in average BMI, proportion of International Classification of Diseases (ICD)-10 codes for obesity, and proportion of weight management recommendations offered by providers among lesbian, bisexual and straight females with BMIs ≥ 30. We classified provider recommendations as definite, possible, and absent. Multivariable median (BMI outcome only) or logistic regression was used to evaluate the associations between sexual orientation and each of the following outcomes: BMI, receipt of obesity diagnosis, and weight management recommendations.

**Results:**

There were no significant differences in BMI, receipt of obesity diagnoses, or weight management recommendations between lesbian, bisexual, and straight females with BMIs ≥ 30. However, only about half the patients with BMIs ≥ 30, regardless of sexual orientation, received a weight management recommendation as recommended by the United States Preventive Services Task Force (USPSTF) guidelines.

**Conclusion:**

We did not observe disparities in BMI, receipt of obesity diagnoses, or receipt of weight management recommendations between sexual orientation minority and heterosexual females among this sample from an urban population of patients receiving care in a university medical system. However, provider recommendation for weight management was suboptimal in all the groups.

**Supplementary Information:**

The online version contains supplementary material available at 10.1186/s12905-021-01585-x.

## Background

Previous research indicates that lesbian and bisexual females have significantly higher odds of being obese compared to their straight counterparts. This may increase their risk of serious illnesses including cancer, cardiovascular, and other diseases [1–19]. In addition, little is known about whether provider diagnosis and recommendation for weight management vary across these populations. However, the United States Preventive Services Task Force (USPSTF) provides evidence-based preventive guidelines for medical providers in regard to the prevention of obesity related disease. These guidelines recommend that “clinicians offer or refer adults with a body mass index of 30 or higher to intensive, behavioral interventions” [20].

Additionally, health care providers have explicit and implicit preferences for straight people in comparison to lesbian or gay people [21]. However, less research exists depicting how this preference affects quality of patient care [21]. Understanding whether obese lesbian and bisexual individuals are less likely to receive a diagnosis of obesity and provider recommendation for weight management, possibly due to factors like implicit bias, may help to identify a potential contributing factor to their poor outcomes due to obesity-related conditions.

## Methods

### Study design

The study has been reviewed and approved by the Institutional Review Board (IRB) at the University of Pennsylvania and was conducted in accordance with all applicable University of Pennsylvania human subjects research requirements as well as applicable federal regulations. This study was reviewed and deemed exempt by the IRB at the University of Pennsylvania. The study met eligibility criteria for IRB review exemption authorized by 45 CFR 46.104, category 4. As part of the exemption determination, a waiver of the Health Insurance Portability and Accountability Act (HIPAA) authorization requirement was granted as authorized by 45 CFR 164.512. The HIPAA is a nationwide act that serves to protect an individual’s health information. An expedited review procedure was used for the HIPAA authorization waiver because the research involves no more than minimal risk to the privacy of the individuals who are the subject of the protected health information for which use or disclosure is being sought. Informed consent was waived since this was a secondary database analysis.

We performed a cross-sectional study of cisgender female patients with BMIs of ≥ 30 who were seen at internal medicine clinics in an urban academic medical center at the University of Pennsylvania between January 1, 2019 and December 31, 2019. BMI was calculated with height and weight measured by providers. The inclusion criteria were age 18–89, cisgender female, BMI of  ≥ 30, and  self-reported sexual orientation as lesbian, bisexual or straight. The exclusion criterion was a diagnosis of pregnancy. Of the 3,782 cisgender female patients who met inclusion and exclusion criteria, 49 patients identified as bisexual, 85 patients identified as lesbian, and 3648 patients identified as straight. During analysis, we used a 1:3 matched control for the bisexual group and a separate 1:3 matched control for the lesbian group. For every one patient who identified as lesbian or bisexual, we analyzed three patients who identified as straight. Out of the 3,648 patients who identified as straight, we used simple random sampling to select 147 patients of the 3,648 patients who identified as straight to compare to the 49 patients who identify as bisexual. Then, we used simple random sampling to select another 255 patients of the 3,648 patients who identified as straight to compare to the 85 patients who identified as lesbian. The dataset had one patient who identified as bisexual with a missing value for obesity and one patient who identified as straight with a missing value for marital status so we only analyzed 48 patients who identified as bisexual and 146 patients who identifed as straight, instead of 49 and 147 respectively.

We ran 2 analyses for weight management recommendation. First, a yes recommendation was defined only as having a definite weight management recommendation documented in the electronic medical record. If the patient did not have documentation of a definite weight management recommendation, then they were counted as not having any recommendation. Then, we reran our model combining definite and possible recommendations for the weight management recommendation variable. If the patient did not receive either a definite or possible recommendation, then they were counted as not having a recommendation.

### Data collection

We collected total number of visits, provider type (Doctor of Medicine (MD), Doctor of Osteopathic Medicine (DO), Certified Registered Nurse Practitioner (CRNP)), BMI, race, ethnicity, age at visit, sexual orientation, gender, marital status, and presence of a diagnosis of obesity, hypertension, dyslipidemia, pre-diabetes, and type 2 diabetes based on ICD-10 codes listed in Additional file 1: Supplementary Appendix SA1. Then, we conducted a manual chart abstraction for provider recommendation for weight management. Two researchers separately read each potential recommendation and then discussed the classification of recommendation. We classified weight management recommendations as definite yes, possible yes, or absent according to qualities of the recommendation outlined in Additional file 1: Supplementary Table ST1. For the patients who had more than 1 visit in the year, we selected the visit that had a definite yes recommendation. Additional details about selecting a visit with a definite yes recommendation can be found in Additional file 1: Supplementary Methods SM1.

### Statistical analysis

Patient and medical visit characteristics were summarized with descriptive statistics and compared between either lesbian or bisexual patients and straight patients using Fisher’s exact test for categorical variables and Wilcoxon rank-sum test for continuous variables. Multivariable median regression was used to evaluate the association between sexual orientation and body mass index and included variables that were identified a priori as potential confounders and included: age, race, ethnicity, marital status, body mass index (not included for the body mass index outcome), history of pre-diabetes or type 2 diabetes, history of hypertension, history of dyslipidemia, history of obesity (included for weight management recommendation outcome only), total number of medical visits, and medical provider type. Multivariable logistic regression was used to evaluate the associations between sexual orientation and each of the following binary outcomes: (i) obesity diagnosis and (ii) weight management recommendation. Multivariable logistic regression models included variables that were statistically significantly different between the lesbian or bisexual groups and their respective straight comparison groups. Statistical significance was determined by two-sided p values at p < 0.05. Statistical analyses were performed using SAS version 9.4 and Stata version 16.1.

## Results

There were 3872 people who met all inclusion and exclusion criteria, including sexual orientation. 3334 people met all other inclusion and exclusion criteria except did not list their sexual orientation so had to be excluded from the study. Thus, 46.27% of patients were excluded due to not listing their sexual orientation.

The demographics of the patients are listed in Table 1. We compared females who identify as lesbian or bisexual to females who identify as straight and found no significant differences in demographic characteristics except the following: (1) Patients who identify as lesbian were more likely to be single and were less likely to be married (p = 0.01) than patients who identify as straight; (2) Patients who identify as lesbian were more likely to be white than patients who identify as straight (p = 0.03); (3) Patients who identify as bisexual were younger (p < 0.00) and less likely to be diagnosed with hypertension (p = 0.00) and dyslipidemia (p = 0.00) than patients who identify as straight; and (4) Patients who identify as bisexual were more likely to be single and were less likely to be married (p < 0.00) than patients who identify as straight.

**Table 1:** Patient demographics

Variable	Lesbian (n = 85)	Straight (n = 255)	p-value	Bisexual (n = 48)	Straight (n = 146)	p-value
	N (%) or mean (SD^1^) and median (IQR^2^)	N (%) or mean (SD) and median (IQR)		N (%) or mean (SD) and median (IQR)	N (%) or mean (SD) and median (IQR)	
Age (years)	50.81 (14.22) 50 (39–62)	53.07 (15.30) 54 (41–65)	0.22^9^	36.35 (12.62) 35 (27–42.5)	55.54 (15.05) 56.5 (45–67)	< 0.00^9^
**Race**			0.03^8^			1.00^8^
White	39 (45.88)	81 (31.76)		16 (33.33)	50 (34.25)	
Black	45 (52.94)	162 (63.53)		30 (62.50)	91 (62.33)	
Asian/other	1 (1.18)	12 (4.71)		2 (4.17)	5 (3.42)	
Other	0	5 (1.96)		1 (2.08)	1 (1.37)	
**Ethnicity**			0.83^8^			0.14^8^
Non-Hispanic Non-Latino	84 (98.82)	246 (96.47)		44 (91.67)	142 (97.26)	
Hispanic Latino	1 (1.18)	7 (2.75)		4 (8.33)	3 (2.05)	
Patient declined	0	2 (0.78)		0	1 (0.68)	
**Marital status**			0.01^8^			< 0.00^8^
Single	47 (55.29)	106 (41.57)		38 (79.17)	53 (36.30)	
Married/partner (including domestic partner)	33 (38.82)	103 (40.39)		9 (18.75)	52 (35.62)	
Separated/other (including divorced, widowed, other)	5 (5.88)	46 (18.04)		1 (2.08)	41 (28.08)	
**Provider type**			0.53^8^			0.06^8^
MD^3^/DO^5^	66 (77.65)	207 (81.18)		45 (93.75)	119 (81.51)	
CRNP^4^	19 (22.35)	48 (18.82)		3 (6.25)	27 (18.49)	
Pre-DM^6^ or Type 2-DM	36 (42.35)	118 (46.27)	0.62^8^	21 (43.75)	71 (48.63)	0.62^8^
Hypertension	37 (43.53)	130 (50.98)	0.26^8^	12 (25.00)	78 (53.42)	0.00^8^
Dyslipidemia	37 (43.53)	108 (42.35)	0.90^8^	8 (16.67)	65 (44.52)	0.00^8^
Obesity	47 (44.29)	127 (49.80)	9.45^8^	27 (56.25)	78 (53.42)	0.74^8^
Total number of visits in the year	2.64 (2.42) 2 (1–3)	2.75 (2.24) 2 (1–3)	0.37^9^	2.52 (1.62) 2 (1–3.5)	2.51 (1.81) 2 (1–3)	0.82^9^
BMI^7^(kg/m^2^)	37.42 (6.64) 35.53 (32.32–40.79)	36.97 (7.08) 34.82 (32.26–39.38)	0.44^9^	37.82 (7.11) 35.49 (31.37–43.74)	36.60 (6.61) 34.79 (31.60–39.82)	0.42^9^

^1^Standard deviation; ^2^Interquartile range; ^3^Doctor of Medicine; ^4^Certified Registered Nurse Practitioner; ^5^Doctor of Osteopathic Medicine; ^6^Diabetes mellitus; ^7^Body Mass Index (kg/m^2^); ^8^Fisher’s Exact; ^9^Wilcoxon rank-sum test

As shown in Table 1, the median (interquartile range) BMIs for the patients who identify as lesbian and straight were 35.53 (32.32–40.79) and 34.82 (32.26–39.38), respectively. The median (interquartile range) BMIs for the patients who identify as bisexual and straight were 35.49 (31.37–43.74) and 34.79 (31.60–39.82), respectively. The unadjusted bivariate analysis showed no statistically significant difference in BMI between the patients who identify as lesbian and straight (p = 0.44) or between patients who identify as bisexual and straight (p = 0.42). As shown in Table 2, there was no statistically significant difference in BMI between patients who identify as lesbian, bisexual, and straight when controlling for age at visit, total number of visits, race, ethnicity, marital status, provider type, and diagnosis of pre or type 2 diabetes, hypertension, or dyslipidemia (p = 0.74 for patients who identify as lesbian and straight and p = 0.67 for patients who identify as bisexual and straight).

**Table 2:** BMI^1^ comparison between patients

Variable	Beta coefficient (lesbian)	p-value (lesbian)	95% confidence interval (lesbian)	Beta coefficient (bisexual)	p-value (bisexual)	95% confidence interval (bisexual)
Age at visit (years)	− 0.14	0.00	− 0.20 to − 0.07	− 0.11	0.09	− 0.24 to 0.02
Total number of visits in the year	0.58	0.00	0.20 to 0.95	0.63	0.12	− 0.17 to 1.43
**Sexual orientation**						
Straight	Reference			Reference		
Lesbian/bisexual respectively	0.30	0.74	− 1.50 to 2.11	− 0.79	0.67	− 4.38 to 2.80
**Race**						
White	Reference			Reference		
Black	0.59	0.54	− 1.32 to 2.50	0.40	0.80	− 2.74 to 3.54
Asian/other	− 1.68	0.43	− 5.91 to 2.54	− 0.47	0.91	− 8.39 to 7.44
**Ethnicity**						
Non-Hispanic Non-Latino	Reference			Reference		
Hispanic Latino	0.21	0.94	− 4.90 to 5.31	1.07	0.77	− 6.09 to 8.22
**Marital status**						
Single	Reference			Reference		
Married/partner/domestic partner	0.44	0.63	− 1.37 to 2.25	0.07	0.97	− 3.30 to 3.45
Separated/divorced/widowed/other	1.22	0.33	− 1.22 to 3.66	− 0.46	0.82	− 4.47 to 3.56
**Provider type**						
MD^2^/DO^4^	Reference			Reference		
CRNP^3^	− 1.60	0.10	− 3.53 to 0.33	− 0.12	0.95	–3.76 to 3.52
Pre-DM^5^ or Type 2-DM	1.49	0.10	− 0.29 to 3.28	3.62	0.02	0.48 to 6.77
Hypertension	1.13	0.22	− 0.69 to 2.95	1.02	0.55	− 2.30 to 4.35
Dyslipidemia	− 0.16	0.87	− 2.10 to 1.77	− 1.45	0.38	− 4.72 to 1.81

^1^Body Mass Index (kg/m^2^); ^2^Doctor of Medicine; ^3^Certified Registered Nurse Practitioner; ^4^Doctor of Osteopathic Medicine ^5^Diabetes Mellitus

Next, we compared the proportion of lesbian, bisexual and straight female patients with BMIs ≥ 30 who received a diagnostic code for obesity. As shown in Table 3, the unadjusted bivariate model showed there was no statistically significant difference in the proportion of patients with BMIs ≥ 30 who received a diagnostic code for obesity between patients who identify as lesbian and straight (p = 0.45) and patients who identify as bisexual and straight (p = 0.74). As shown in Table 4, there was no significant difference in number of patients who received the diagnosis of obesity between patients who identify as lesbian, bisexual, and straight even when controlling for age at visit (bisexual only), race (lesbian only), marital status, provider type (bisexual only), and diagnosis of relevant medical conditions hypertension (bisexual only), or dyslipidemia (bisexual only) (aOR 1.31 95% CI [0.78–2.19], p = 0.30 for patients who identify as lesbian and straight) and (aOR 0.89, 95% CI [0.40–1.98], p = 0.78 for patients who identify as bisexual and straight). Of note as shown in Table 3, 44.71% of patients who identify as lesbian and 50.20% of patients who identify as straight to which they were compared did not receive a diagnosis code of obesity. Similarly, 43.75% of patients who identify as bisexual and 46.58% of patients who identify as straight to which they were compared did not receive a diagnosis code of obesity.

**Table 3:** Bivariate analysis for patients who received a recommendation for weight management and obesity diagnosis

	Sexual orientation				Unadjusted p value (Fisher’s exact)	
	Lesbian (n = 85)	Straight (n = 255)	Bisexual (n = 48)	Straight (n = 146)	Lesbian	Bisexual
**Definite plus possible recommendation**						
No (%)	35.29	35.69	29.17	37.67	1.00	0.30
Yes (%)	64.71	64.31	70.83	62.33		
**Definite yes recommendation**						
No (%)	55.29	51.37	43.75	50.00	0.62	0.51
Yes (%)	44.71	48.63	56.25	50.00		
**Diagnosis code of obesity**						
No (%)	44.71	50.20	43.75	46.58	0.45	0.74
Yes(%)	55.29	49.80	56.25	53.42

**Table 4:** Multivariable analysis of the proportion of patients who received a diagnostic code for obesity

Variable	aOR^4^ (lesbian)	95% confidence interval (lesbian)	p-value (lesbian)	aOR^4^ (bisexual)	95% confidence interval (bisexual)	p-value (bisexual)
Age at visit (years)				0.99	0.96 to 1.01	0.32
**Sexual orientation**			0.30			0.78
Straight	Reference					
Lesbian/bisexual respectively	1.31	0.78 to 2.19		0.89	0.40 to 1.98	
**Race**			0.11			
White	Reference					
Black	1.63	1.00 to 2.67				
Asian/other	0.86	0.26 to 2.82				
**Marital status**			0.69			0.74
Single	Reference					
Married/partner/domestic partner	0.82	0.49 to 1.34		0.97	0.47 to 2.01	
Separated/divorced/widowed/other	1.00	0.52 to 1.92		0.72	0.30 to 1.76	
**Provider type**						0.05
MD^1^/DO^3^	Reference					
CRNP^2^				0.43	0.18 to 0.98	
Hypertension				2.76	1.33 to 5.74	0.01
Dyslipidemia				0.80	0.39 to 1.66	0.55

1Doctor of Medicine; ^2^Certified Registered Nurse Practitioner; ^3^Doctor of Osteopathic Medicine; ^4^Adjusted Odds Ratio

We then compared the proportion of lesbian, bisexual, and straight cisgender female patients who received a provider recommendation for weight management. As shown in Table 3, there was no significant difference between the proportion of definite weight management recommendations received between the patients who identify as lesbian and straight (p = 0.62) or patients who identify as bisexual and straight (p = 0.51). As shown in Table 5, there was no significant difference in proportion of definite weight management recommendations received between the patients who identify as lesbian, bisexual, and straight even when controlling for age at visit (bisexual only), race (lesbian only), marital status, provider type (bisexual only), and diagnosis of hypertension (bisexual only), or dyslipidemia (bisexual only) (aOR 0.81 95% CI [0.49–1.34], p = 0.41 for patients who identify as lesbian and straight) and (aOR 1.44, 95% CI [0.65–3.18], p = 0.36 for patients who identify as bisexual and straight).

**Table 5:** Multivariable analysis of patients who received a definite yes recommendation for weight management

Variable	aOR^4^ (lesbian)	95% confidence interval (lesbian)	p-value (lesbian)	aOR^4^ (bisexual)	95% confidence interval (bisexual)	p-value (bisexual)
**Age at visit (years)**				1.00	0.97 to 1.03	0.96
**Sexual orientation**			0.41			0.36
Straight	Reference					
Lesbian/bisexual respectively	0.81	0.49 to 1.34		1.44	0.65 to 3.18	
**Race**			0.09			
White	Reference					
Black	1.03	0.63 to 1.67				
Asian/other	0.18	0.04 to 0.87				
**Marital status**			0.95			0.34
Single	Reference					
Married/partner/domestic partner	1.03	0.62 to 1.70		0.60	0.29 to 1.21	
Separated/divorced/widowed/other	0.92	0.48 to 1.76		0.85	0.35 to 2.07	
**Provider type**						0.65
MD^1^/DO^3^	Reference					
CRNP^2^				1.21	0.53 to 2.74	
Hypertension				2.06	1.02 to 4.17	0.04
Dyslipidemia				0.97	0.47 to 1.98	0.93

1Doctor of Medicine ^2^Certified Registered Nurse Practitioner ^3^Doctor of Osteopathic Medicine ^4^Adjusted Odds Ratio

As shown in Table 3, there was no significant difference between the proportion of definite and possible provider recommendations received between the patients who identify as lesbian and straight (p = 1.00) and patients who identify as bisexual and straight (p = 0.30). As shown in Table 6, there was no significant difference in proportion of definite or possible weight management recommendations between the patients who identify as lesbian, bisexual, and straight even when controlling for age at visit (bisexual only), race (lesbian only), marital status, provider type (bisexual only), and diagnosis of medical conditions including hypertension (bisexual only), or dyslipidemia (bisexual only) (aOR 0.97, 95% CI [0.57–1.65], p = 0.91 for patients who identify as lesbian and straight) and (aOR 1.52, 95% CI [0.66–3.49], p = 0.32 for patients who identify as bisexual and straight).

**Table 6:** Multivariable analysis of patients who received either a definite yes or possible yes recommendation for weight management

Variable	aOR^4^ (lesbian)	95% confidence interval (lesbian)	p-Value (lesbian)	aOR^4^ (bisexual)	95% confidence interval (bisexual)	p-value (bisexual)
**Age at visit (years)**				1.00	0.97 to 1.02	0.76
**Sexual orientation**			0.91			0.32
Straight	Reference					
Lesbian/bisexual respectively	0.97	0.57–1.65		1.52	0.66–3.49	
**Race**			0.39			
White	Reference					
Black	0.99	0.60–1.65				
Asian/other	0.45	0.14–1.45				
**Marital status**			0.93			0.81
Single	Reference					
Married/partner/domestic partner	0.95	0.57–1.60		0.93	0.45 to 1.93	
Separated/divorced/widowed/other	0.88	0.45–1.72		1.24	0.49 to 3.11	
**Provider type**						0.81
MD^1^/DO^3^	Reference					
CRNP^2^				0.90	0.39 to 2.08	
Hypertension				1.84	0.89 to 3.81	0.10
Dyslipidemia				0.74	0.36 to 1.54	0.42

1Doctor of Medicine; ^2^Certified Registered Nurse Practitioner; ^3^Doctor of Osteopathic Medicine; ^4^Adjusted Odds Ratio

As shown in Table 3, 55.29% of patients who identify as lesbian and 51.37% of patients who identify as straight to which they were compared did not receive a definite recommendation for weight management. Similarly, 43.75% of patients who identify as bisexual and 50.00% of patients who identify as straight to which they were compared did not receive a definite yes recommendation for weight management.

## Discussion

To our knowledge, this is one of the first studies to examine whether sexual orientation minorities who have BMIs ≥ 30 are less likely to receive a diagnosis of obesity and provider recommendation for weight management in their medical records. We found that there is no significant difference in BMI between the patients who identify as lesbian and bisexual when compared to their straight counterparts. We also found that sexual orientation does not impact the proportion of female patients with BMIs of ≥ 30 who receive a diagnosis of obesity. Importantly, we also found that sexual orientation does not impact the proportion of female patients with BMIs of ≥ 30 who receive a weight management recommendation from a provider.

Additionally of note, only approximately half or just over half of all patients with BMIs of ≥ 30 received an obesity diagnosis code and weight management recommendation from their provider. It is clear that BMI needs to be discussed more frequently at office visits as this may facilitate a reduction in prevalence of obesity-related diseases. Similarly, other research notes that obese females have the same amount of time spent at their visits with their primary care provider compared to non-obese females [22]. These considerations are especially important among patients who identify as lesbian and bisexual as previous research shows this population is more likely to be obese compared to their straight counterparts.

It is important to note that Philadelphia is known to be an LGBTQ (lesbian, gay, bisexual, transgender, queer) friendly city and Penn Medicine strongly promotes diversity and inclusion. Penn Medicine has an LGBTQ health program which lists specific providers who identify themselves as LGBTQ friendly providers. It is possible this designation has been successful in serving as a welcoming message to patients of all sexual orientations and it is an important consideration for other medical centers when striving towards equitable medical care. This may contribute to the lack of significant difference in proportion of weight management recommendations, BMI, and diagnoses of obesity between lesbian, bisexual, and straight females with BMIs of ≥ 30.

## Limitations

This study has several limitations. First, the generalizability of the study may be limited due to the small sample size of the sexual orientation minorities with BMIs of ≥ 30. One reason for the small sample size may be because patients who identify as lesbian and bisexual did not choose to disclose their sexual orientation due to fear of healthcare discrimination [23]. 46.27% of patients who otherwise met inclusion and exclusion criteria were excluded from our study due to not listing their sexual orientation. Additionally, education level and household income were not listed for many patients in the electronic medical records which are factors that influence the incidence of obesity [24]. Another limitation is that this is a single department, single institution study and it remains to be demonstrated whether the experiences are the same in other institutions in different geographic areas that may not be as LGBTQ friendly. An additional potential limitation is non-differential under-reporting. It is possible that providers actually counseled patients about weight management, but did not record it in the chart for reasons such as time constraints. In addition, menopausal and parity status were not available for every patient which are both factors that may impact BMI and therefore impact our results. Lastly, having two different straight control groups to compare to the patients who identify as bisexual and lesbian may impact our results.

## Conclusion

This research provides some reassurance that there may not be inequities in BMI, receiving a diagnosis of obesity, and receiving a recommendation for weight management in obese lesbian and bisexual female patients with BMIs ≥ 30. In addition, this paper highlights that providers are not documenting the diagnosis of obesity in the medical record and providing a weight management recommendation to approximately half of their patients with BMIs of ≥ 30 despite USPSTF guidelines. This has the potential to propagate the obesity epidemic and may contribute to the premature death of many individuals with BMIs of ≥ 30. As such, there is a need for interventions that help increase providers diagnosis and management of obesity as this may facilitate a reduction in prevalence of obesity-related diseases. These considerations are especially important among patients who identify as lesbian and bisexual as previous research shows this population is more likely to be obese compared to their straight counterparts. Future studies in diverse departments and institutions as well as follow up studies that determine the effectiveness of weight management recommendations are warranted. Additionally, studies aimed at identifying unique barriers the LGBTQ community may face in losing weight should be considered.

## Supplementary Information

**Additional file 1**. Supplementary Table ST1. Supplementary Appendix SA1. Supplementary Methods SM1.

## Data Availability

The datasets generated and analyzed during the current study are not publicly available because my original IRB protocol did not propose publicly posting de-identified data and thus, we do not have permission to do that. However, the datasets used and analyzed during the current study are available from the corresponding author on reasonable request.

## Abbreviations

aOR: Adjusted odds ratio
BMI: Body Mass Index
CI: Confidence Interval
CRNP: Certified Registered Nurse Practitioner
DO: Doctor of Osteopathic Medicine
HIPAA: Health Insurance Portability and Accountability Act
ICD: International Classification of Diseases
IRB: Institutional Review Board
LGBTQ: Lesbian, gay, bisexual, transgender, queer
MD: Doctor of Medicine
OR: Odds ratio
SA1: Supplementary Appendix
SM1: Supplementary Methods
ST1: Supplementary Table
USPSTF: United States Preventive Services Task Force
