# Correction: The association between sexual orientation, BMI, obesity diagnosis, and provider recommendation for weight management

**DOI:** 10.1186/s12905-022-02047-8

**Published:** 2022-11-15

**Authors:** Kristen M. Wolfgang, Junko Takeshita, Robert Fitzsimmons, Carmen E. Guerra

**Affiliations:** 1 University of Pennsylvania Perelman School of Medicine, 3400 Civic Center Blvd, Philadelphia, PA 19104 USA; 2 Department of Dermatology and Department of Biostatistics, Epidemiology and Informatics, University of Pennsylvania Perelman School of Medicine, 3400 Civic Center Blvd, Philadelphia, PA 19104 USA; 3 Department of Dermatology, University of Pennsylvania Perelman School of Medicine, 3400 Civic Center Blvd, Philadelphia, PA 19104 USA; 4 Division of General Internal Medicine, University of Pennsylvania Perelman School of Medicine, 423 Guardian Drive, Philadelphia, PA 19104 USA

**Correction: BMC Women’s Health 22, 19 (2022)**

**https://doi.org/10.1186/s12905-021-01585-x**

Following publication of the original article [1], the corresponding email id has changed from kristen.wolfgang@pennmedicine.upenn.edu to kwolfgang8@gmail.com

The original article has been corrected.
