# Supplementary material for: The association between sexual orientation, BMI, obesity diagnosis, and provider recommendation for weight management
Source: BMC Womens Health. 2022 Jan 26;22:19. doi: 10.1186/s12905-021-01585-x (PMC8793187; doi:10.1186/s12905-021-01585-x)
Supplement: Supplementary file 1 — Additional file 1. Supplementary Table ST1. Supplementary Appendix SA1. Supplementary Methods SM1. [file 12905_2021_1585_MOESM1_ESM.docx]

| **Supplementary Table ST1: Classification of Weight Management Recommendations** | | |
| --- | --- | --- |
| **Definite evidence of Weight management recommendation** | **Possible weight management recommendation** | **No weight management recommendation** |
| 1. Encounter notes indicated there was a discussion about weight or dietary changes (in regard to any condition-obesity or other obesity related conditions such as diabetes)  2. Encounter notes indicated the provider discussed weight, diet, or exercise  3. Encounter notes indicated there was a discussion in which the doctor discussed/offered or prescribed weight loss options- surgery or pharmacotherapy  4. Provider noted that they counseled patient on weight or diet in regard to obesity/BMI/or other weight-related condition (GERD, yeast infections, diabetes, hypertension)  5. Presence of an order/referral to nutritionist/obesity/weight management/ GI surgery /bariatric surgery/behavioral health  6. Patient care instructions stated start exercise, make diet changes, provide exercising resources or tips, nutrition/diet resources such as fitness and/or diet apps or programs or recipes  7. Created/listed goals in regard to diet, exercise, and/or weight loss  8. Provider educated patient about importance of losing weight/diet/exercise or about healthy lifestyle modifications  9. Provider encouraged patient to continue current/ongoing regimen losing weight, making certain diet changes, pharmacotherapy for weight loss, seeing dietician, seeing trainer, going to the gym/exercising, lifestyle modifications in regard to obesity (including phrasing like will continue to work on diet)  10. Provider encouraged patient to start regimen losing weight, making certain diet changes, seeing dietician, seeing trainer, going to the gym/exercising, lifestyle modifications (healthy lifestyle, lifestyle reviewed) in regard to obesity/other conditions/unspecified  11. Encounter note indicated discussion of prescriptions for weight loss pharmacotherapies (encourage- ment/recommendations/modifications or any further comments)  12. Documentation of telephone call or voicemail in which the provider noted a discussion of lifestyle modifications | 1. Doesn’t meet anything in “definite” criteria  2. Encounter note documented existing diet or exercise plan in history section addressing obesity or other obesity-related conditions (no encouragement or other comments noted)  3. Provider documented would reassess weight/diet at next visit  4. Note acknowledged that diabetes is diet-controlled  5. Note acknowledged patient is working with another person or agency for weight loss/nutrition (endocrinology, dietician, MANNA, IMPACT)  6. Provider stated patient has an existing weight loss plan/is actively working on weight loss but without encouragement  7. Provider documented previous prescriptions for weight loss pharmacotherapy but without encouragement/recommendations/modifications or any further comments) | 1. Provider asked patient to record daily weights (or call office if they gain weight) in regard to heart failure, pulmonary hypertension or coronary artery disease  2. Provider documented patient’s weight loss, gain, or stability in history  3.Data from last  visit written beneath weight readings from previous encounters  4. Provider documented giving patient specific exercises for acute issue NOT exercise for the goal of weight loss, but rather to manage that condition (for example, pelvic floor exercises for urinary incontinence or exercises for knee osteoarthritis)  5. Assessment of exercise capacity in a pre-op exam  6. No mention of weight, diet, exercise, or nutrition in patient encounter notes or patient instructions for that visit and no definite or possible yes recommendations  8. Documentated discussion of weight gain specifically in relation to heart failure water weight  9. When it was unclear what provider documented patient is up to date on preventive healthcare and did not note if they discussed weight, diet, or exercise with the patient |

**Supplementary Appendix SA1**

1. Hyperlipidemia/Dyslipidemia diagnosis includes:

- Dyslipidemia E78.5
- Familial Hypercholesterolemia E78.01
- HLD (hyperlipidemia) E78.5
- Hypercholesterolemia E78.00
- Hyperlipidemia E78.00
- Hyperlipidemia E78.2
- Hyperlipidemia E78.5
- Hyperlipidemia LDL goal <130 E78.5
- Hyperlipidemia LDL goal <70 E78.5
- Hyperlipidemia NEC/NOS - LDL goal < 70 E78.5
- Hyperlipidemia on statin
- Hyperlipidemia with target LDL less than 100 E78.5
- Hyperlipidemia with target LDL less than 70 E78.5
- Hyperlipidemia, unspecified hyperlipidemia type
- Hypertriglyceridemia E78.1
- Lipidemia E78.5
- Mixed hyperlipidemia LDL<100 E78.2
- Mixed hyperlipidemia, E78.2
- Other and unspecified hyperlipidemia E78.2
- Other and unspecified hyperlipidemia E78.5
- Other hyperlipidemia E78.49
- Pure hypercholesteremia E78.00
- Pure hypercholesterolemia with target low density lipoprotein (LDL) cholesterol less than 70 mg/dL E78.00
- Pure hypertriglyceridemia E78.1

*Excluded lipidosis

1. Hypertension diagnosis includes:
   - - Benign essential HTN I10
     - Benign Hypertension
     - Benign hypertension with CKD (chronic kidney disease) stage III
     - Elevated blood pressure reading with diagnosis of hypertension
     - Essential hypertension diagnosed age 30's I10
     - Essential hypertension I10
     - Essential hypertension with goal blood pressure less than 130/85
     - Essential hypertension with goal blood pressure less than 140/90
     - Essential hypertension, benign I10
     - Essential hypertension, malignant
     - High blood pressure I10
     - HTN (hypertension) I10
     - HTN (hypertension)/ Dx ~ 1992 I10
     - HTN, goal below 130/80 I10
     - HTN, goal below 140/90
     - Hypertension I10
     - Hypertension secondary to other renal disorders I15.1
     - Hypertension, unspecified
     - Hypertension, unspecified type
     - Labile hypertension R09.89
     - Malignant Hypertension I10
     - Unspecified essential hypertension I 10

*Excluded elevated BP

*Excluded gestational hypertension, hypertension peripartum

*Excluded any diagnoses in which it was unclear if the hypertension occurred before or during pregnancy

1. Obesity Diagnosis includes:

- (Body mass index (BMI) 32.0-32.9, adult) Z68.32
- (Body mass index (BMI) 33.0-33.9, adult Z68.33
- (Body mass index (BMI) 39.0-39.9, adult Z68.39
- Adult BMI 37.0-37.9 kg/sq m
- BMI 32.0-32.9, adult
- BMI 34.0-34.9, adult
- BMI 35.0-35.9, adult
- BMI 38.0-38.9 adult E68.38
- BMI 39.0-39.9, adult Z68.39
- BMI 40.0-44.9, adult
- BMI 40.0-44.9, adult Z68.41 (BMI 40-45.9)
- BMI 50.0-59.9, adult (CMS-HCC) Z68.43x
- Body mass index (BMI) of 45.0-49.9 in adult Z68.42
- Body mass index 36.0-36.9, adult Z68.36
- Class 1 obesity with serious comorbidity and body mass index (BMI) of 32.0 to 32.9 in adult
- Class 1 obesity without serious comorbidity with body mass index (BMI) of 30.0 to 30.9 in adult, unspecified obesity type
- Class 2 obesity due to excess calories with body mass index (BMI) of 35.0 to 35.9 in adult E66.09
- Class 2 obesity with body mass index (BMI) of 36.0 to 36.9 in adult, unspecified obesity type, unspecified whether serious comorbidity present
- Class 2 obesity with body mass index (BMI) of 37.0 to 37.9 in adult E66.9
- Class 2 obesity with body mass index (BMI) of 38.0 to 38.9 in adult, unspecified obesity type, unspecified whether serious comorbidity present
- Class 2 obesity without serious comorbidity with body mass index (BMI) of 36.0 to 36.9 in adult E66.9
- Class 2 obesity without serious comorbidity with body mass index (BMI) of 39.0 to 39.9 in adult, unspecified obesity type
- Class 2 severe obesity due to excess calories with serious comorbidity and body mass index (BMI) of 37.0 to 37.9 in adult (CMS-HCC) E66.01
- Class 3 obesity due to excess calories with body mass index (BMI) of 40.0 to 44.9 in adult IMO0001
- Class 3 obesity without serious comorbidity with body mass index (BMI) of 40.0 to 44.9 in adult IMO0001
- Class 3 severe obesity due to excess calories with serious comorbidity and body mass index (BMI) of 40.0 to 44.9 in adult (CMS-HCC) E66.01
- Class 3 severe obesity in adult (CMS-HCC) E66.01
- Class 3 severe obesity with body mass index (BMI) of 40.0 to 44.9 in adult (CMS-HCC)
- Class 3 severe obesity with body mass index (BMI) of 40.0 to 44.9 in adult, unspecified obesity type, unspecified whether serious comorbidity present
- Class 3 severe obesity with body mass index (BMI) of 45.0 to 49.9 in adult, unspecified obesity type, unspecified whether serious comorbidity present
- Class 3 severe obesity with body mass index (BMI) of 50.0 to 59.9 in adult, unspecified obesity type, unspecified whether serious comorbidity present
- Class 3 severe obesity with serious comorbidity and body mass index (BMI) of 60.0 to 69.9 in adult, unspecified obesity type
- Class 3 severe obesity without serious comorbidity with body mass index (BMI) of 40.0 to 44.9 in adult (CMS-HCC) E66.01
- Class 3 severe obesity without serious comorbidity with body mass index (BMI) of 45.0 to 49.9 in adult, unspecified obesity type (CMS-HCC)
- Diabetes mellitus type 2 in obese
- Morbid obesity due to excess calories (CMS-HCC) E66.1
- Morbid Obesity E66.01
- Morbid obesity with BMI of 45.0-49.9, adult
- Morbid obesity with BMI of 50.0-59.9, adult (CMS-HCC)
- Morbid obesity with BMI of 60.0-69.9, adult (CMS-HCC)
- Morbid obesity, BMI 50 E66.9
- Morbid obesity, unspecified obesity type (CMS-HCC) E66.01
- Non morbid obesity E66.9
- Obese E66.9
- Obesity (BMI 30-39.9) E66.9
- Obesity (BMI 30.0-34.9) E66.9
- Obesity (BMI 35.0-39.9 without comorbidity)
- Obesity due to excess calories with serious comorbidity E66.9
- Obesity E66.9
- Obesity hypoventilation syndrome
- Obesity with body mass index greater than 30
- Obesity, BMI >40 E66.01
- Obesity, Class I, BMI 30-34.9
- Obesity, Class I, BMI 30-34.9
- Obesity, Class I, BMI 30-34.9 E66.9
- Obesity, Class III, BMI 40-49.9 (morbid obesity) (CMS-HCC)
- Obesity, Class III, BMI 40-49.9 (morbid obesity) (CMS-HCC) E66.01
- Obesity, morbid (more than 100 lbs over ideal weight or BMI > 40) (CMS-HCC)
- Obesity, morbid, BMI 50 or higher (CMS-HCC) E66.01
- Obesity, unspecified classification, unspecified obesity type, unspecified whether serious comorbidity present
- Obesity, unspecified E66.9
- Overweight and obesity (278.0)
- Severe obesity (BMI 35.0-39.9) with comorbidity

*Excluded “obesity complicating pregnancy, childbirth, or puerperium, antepartum” and other diagnoses regarding obesity and pregnancy

*Excluded “excess weight”

1. Pre or type 2 diabetes includes:

- Background diabetic retinopathy(362.01) E11.3299
- Controlled diabetes mellitus type II without complication (CMS-HCC) E11.9
- Controlled type 2 diabetes mellitus without complication, with long-term current use of insulin (CMS-HCC) E11.9
- Diabetes mellitus
- Diabetes mellitus due to underlying condition with chronic kidney disease, without long-term current use of insulin, unspecified CKD stage (CMS-HCC)
- Diabetes mellitus due to underlying condition with severe nonproliferative diabetic retinopathy with macular edema, bilateral (CMS-HCC) E08.3413
- Diabetes mellitus due to underlying condition, controlled, with other circulatory complication, without long-term current use of insulin E08.59
- Diabetes mellitus type 2 in obese (CMS-HCC) E11.69
- Diabetes mellitus type 2, controlled (CMS-HCC) E11.9
- Diabetes mellitus type 2, diet-controlled
- Diabetes mellitus type 2, noninsulin dependent (CMS-HCC) E11.9
- Diabetes mellitus type 2, uncomplicated (CMS-HCC) E11.9
- Diabetes mellitus type II, uncontrolled (CMS-HCC) E11.65
- Diabetes mellitus with background retinopathy (CMS-HCC) E11.3299
- Diabetes mellitus with complication (CMS-HCC) E11.8
- Diabetes mellitus with diabetic nephropathy (CMS-HCC) E11.21
- Diabetes mellitus with ESRD (end-stage renal disease)
- Diabetes mellitus with hemoglobin A1c goal of 7.0%-8.0%
- Diabetes mellitus with renal manifestation (CMS-HCC)
- Diabetes mellitus without complication (CMS_HCC) E11.9
- Diabetes mellitus, controlled
- Diabetes mellitus, type 2 (CMS-HCC) E11.9
- Diabetes type 2, controlled (CMS-HCC) E11.9
- Diabetes type 2, uncontrolled (CMS-HCC) E11.65
- Diabetic foot (CMS-HCC) E11.8
- Diabetic macular edema (CMS-HCC)
- Diabetic mononeuropathy associated with diabetes mellitus due to underlying condition (CMS-HCC) E08.41
- Diabetic neuropathy (CMS-HCC) E11.40
- Diabetic neuropathy associated with type 2 diabetes mellitus (CMS-HCC) E11.40
- Diabetic polyneuropathy E11.42
- Diet-controlled diabetes mellitus (CMS-HCC) E11.9
- DM (diabetes mellitus) (CMS-HCC) E11.9
- DM (diabetes mellitus) type II uncontrolled with eye manifestation (CMS-HCC) E11.39
- DM (diabetes mellitus), type 2 with renal complications (CMS-HCC) E11.29
- DM (diabetes mellitus), type 2, uncontrolled (CMS-HCC) E11.65
- DM type 2, goal HbA1c < 7%
- DM type 2, goal HbA1C <7.5%
- DM type 2, not at goal (CMS-HCC) E11.9
- Moderate nonproliferative diabetic retinopathy of both eyes associated with type 2 diabetes mellitus, macular edema presence unspecified
- Noncompliance with diabetes treatment
- Poorly controlled type 2 diabetes mellitus (CMS-HCC)
- Pre-diabetes - 1/14 5.7% R73.03
- Pre-diabetes (12/2017 A1c = 5.9) R73.03
- Pre-diabetes R73.03
- Proliferative diabetic retinopathy (362.02) E11.3599
- Retinopathy, diabetic background E11.3299
- Type 1 diabetes mellitus with hyperglycemia, with long-term current use of insulin (CMS-HCC) E10.65
- Type 2 diabetes mellitus treated without insulin (CMS-HCC) E11.9
- Type 2 diabetes mellitus with complication
- Type 2 diabetes mellitus with complication, with long-term current use of insulin
- Type 2 diabetes mellitus with complication, without long-term current use of insulin
- Type 2 diabetes mellitus with diabetic chronic kidney disease (CMS-HCC) E11.22
- Type 2 diabetes mellitus with diabetic nephropathy (CMS-HCC) E11.21
- Type 2 diabetes mellitus with diabetic nephropathy, with long-term current use of insulin (CMS-HCC)
- Type 2 diabetes mellitus with diabetic neuropathy, with long-term current use of insulin
- Type 2 diabetes mellitus with diabetic neuropathy, without long-term current use of insulin (CMS-HCC) E11.40
- Type 2 diabetes mellitus with diabetic polyneuropathy (CMS-HCC) E11.42
- Type 2 diabetes mellitus with diabetic polyneuropathy, with long-term current use of insulin
- Type 2 diabetes mellitus with diabetic polyneuropathy, without long-term current use of insulin (CMS-HCC) E11.42
- Type 2 diabetes mellitus with hemoglobin A1c goal of less than 7.0% (CMS-HCC)
- Type 2 diabetes mellitus with hyperglycemia, with long-term current use of insulin (CMS-HCC) E11.65
- Type 2 diabetes mellitus with hyperglycemia, without long-term current use of insulin
- Type 2 diabetes mellitus with mild nonproliferative retinopathy without macular edema, unspecified laterality, unspecified whether long term insulin use
- Type 2 diabetes mellitus with hyperosmolarity without coma, with long-term current use of insulin
- Type 2 diabetes mellitus with mild nonproliferative retinopathy, without long-term current use of insulin (CMS-HCC) E11.3299
- Type 2 diabetes mellitus with mild nonproliferative retinopathy, without long-term current use of insulin, macular edema presence unspecified, unspecified laterality (CMS-HCC)
- Type 2 diabetes mellitus with other specified complication, without long-term current use of insulin
- Type 2 diabetes mellitus with peripheral autonomic neuropathy (CMS-HCC) E11.43
- Type 2 diabetes mellitus with polyneuropathy
- Type 2 diabetes mellitus with renal manifestations (CMS-HCC) E11.29
- Type 2 diabetes mellitus with retinopathy without macular edema, without long-term current use of insulin, unspecified laterality, unspecified retinopathy severity
- Type 2 diabetes mellitus with retinopathy without macular edema, without long-term current use of insulin, unspecified laterality, unspecified retinopathy severity
- Type 2 diabetes mellitus with stage 3 chronic kidney disease, with long-term current use of insulin (CMS-HCC) E11.22
- Type 2 diabetes mellitus without complication (CMS-HCC) E11.9
- Type 2 diabetes mellitus without complication, with long-term current use of insulin (CMS-HCC)
- Type 2 diabetes mellitus without complication, without long-term current use of insulin (CMS-HCC) E11.9
- Type 2 diabetes with complication, s/p CVA E11.8
- Type 2 diabetes with stage 3 chronic kidney disease GFR 30-59 (CMS-HCC) E11.22
- Type 2 diabetes without retinopathy E11.9
- Type 2 diabetes, controlled, with peripheral neuropathy (CMS-HCC) E11.42
- Type 2 diabetes, diet controlled E11.69
- Type 2 diabetes, uncontrolled, with neuropathy (CMS-HCC) E11.40
- Type 2 DM on Insulin E11.40
- Type II diabetes mellitus (CMS-HCC)
- Type II diabetes mellitus with renal manifestations, uncontrolled (CMS-HCC) E11.29
- Type II diabetes mellitus, well controlled
- Type II or unspecified type diabetes mellitus without mention of complication, not stated as uncontrolled
- Uncontrolled diabetes mellitus type 2 without complications (CMS-HCC) E11.65
- Uncontrolled diabetes mellitus with diabetic nephropathy  (primary encounter diagnosis)
- Uncontrolled type 2 diabetes mellitus with complication, without long-term current use of insulin
- Uncontrolled type 2 diabetes mellitus with diabetic nephropathy, with long-term current use of insulin (CMS-HCC)
- Uncontrolled type 2 diabetes mellitus with hyperglycemia (CMS-HCC) E11.65
- Uncontrolled type 2 diabetes mellitus with microalbuminuria (CMS-HCC) E 11.29
- Uncontrolled type 2 diabetes mellitus with peripheral neuropathy (CMS-HCC) E11.42
- Uncontrolled type 2 diabetes mellitus without complication, with long-term current use of insulin
- Uncontrolled type 2 diabetes mellitus without complication, without long-term current use of insulin E11.65
- Uncontrolled type 2 diabetes with renal manifestation
- Uncontrolled type 2 diabetes with stage 5 chronic kidney disease GFR <15 (CMS-HCC) E11.2
- Uncontrolled type II diabetes mellitus with nephropathy (CMS-HCC) E11.21
- Uncontrolled type II diabetes mellitus with polyneuropathy
- Uncontrolled type II diabetes mellitus with proliferative retinopathy (CMS-HCC) E11.3599
- Well controlled diabetes mellitus
- Well controlled type 2 diabetes mellitus (CMS-HCC)

**Supplementary Methods SM1**

If there were multiple visits with a definite yes, we used a random number generator to select a random visit with a definite yes to analyze. If the patient never had a visit with a definite yes recommendation, we selected the visit with a possible yes. If there were multiple visits with a possible yes, we used a random number generator to select a random visit with a possible yes to analyze. If the patient did not have a definite yes or possible yes, then we selected the visit without any recommendation. If there were multiple visits without a recommendation, we used a random number generator to select a random visit without a recommendation to analyze. Even if the patient did not have a diagnosis of obesity, hypertension, dyslipidemia, pre-diabetes, or diabetes at the visit we randomly selected, they were still counted as having the diagnosis if they were diagnosed with it at any of their other visits. To be categorized as having a recommendation, the patient either had a definite yes or possible yes recommendation. Note that definite yes took precedence over possible yes when both classifications were present in the same encounter notes.
